# Presynaptic NR2A-Containing NMDARs Are Required for LTD between the Amygdala and the Perirhinal Cortex: A Potential Mechanism for the Emotional Modulation of Memory?^1^,^2^,^3^

**DOI:** 10.1523/ENEURO.0046-14.2015

**Published:** 2015-03-23

**Authors:** Michael D. Laing, Zafar I. Bashir

**Affiliations:** 1 Neuroscience and Mental Health Research Institute, Hadyn Ellis Building, Cathays, Cardiff, CF24 4HQ, United Kingdom; 2 School of Physiology and Pharmacology, Bristol University, Bristol, BS8 1TD, United Kingdom

**Keywords:** amygdala, emotion, memory, perirhinal, plasticity, transmission

## Abstract

Visual recognition memory relies on long-term depression-like mechanisms within the perirhinal cortex and the activation of the lateral amygdala can enhance visual recognition memory. How the lateral amygdala regulates recognition memory is not known, but synaptic plasticity at amygdala−perirhinal synapses may provide a mechanism for the emotional enhancement of recognition memory. In this study, we investigate the mechanisms of long-term depression (LTD) at the amygdala−perirhinal synapse in male Lister Hooded rats. We demonstrate that LTD at this input relies on NR2A-containing NMDARs, located presynaptically. Therefore, the underlying mechanisms of LTD, at the amygdala−perirhinal input, which may regulate the emotional context for recognition memory, are different to previously described postsynaptic NR2B−NMDAR mechanisms of intraperirhinal LTD that subserve recognition memory.

## Abstract (precis)

Emotional events are better remembered than emotionally neutral events. The ability of memory enhancement by emotion is dependent on amygdala-mediated alterations of synaptic activity.

## Significance Statement

Emotional events are better remembered than emotionally neutral events. The ability of memory enhancement by emotion is dependent on amygdala-mediated alterations of synaptic activity. The amygdala has robust connections with perirhinal cortex, modulating synaptic transmission and plasticity within this region. The perirhinal cortex is necessary for visual object recognition, relying on long-term depression for memory. Despite the importance of emotion to modulate memory in perirhinal cortex, the mechanisms are unknown. We provide the first demonstration of the mechanisms underlying LTD at this synapse, dependent on presynaptically located NR2A-containing NMDARs. We show a role for NMDARs in amygdala−perirhinal plasticity, and that the mechanisms described here are different to those of amygdala−perirhinal LTP (NMDAR-independent) and intraperirhinal plasticity (NMDAR-dependent), both of which are induced postsynaptically.

## Introduction

The familiarity discrimination component of visual recognition memory requires the perirhinal cortex, as demonstrated in both macaque (Gaffan and Murray, 1992; Fahy et al., 1993; Suzuki et al., 1993; Suzuki, 1996; Murray and Bussey, 1999) and rodent (Aggleton et al., 1997; Ennaceur and Aggleton, 1997; Ennaceur et al., 1997; Dix and Aggleton, 1999; Winters et al., 2004) models, relying on lasting decrements in neuronal activation (Zhu and Brown, 1995; Brown and Xiang, 1998). There is a clear evidence that synaptic plasticity in the perirhinal cortex may provide the cellular mechanisms underlying recognition memory (Warburton et al., 2003; Griffiths et al., 2008; Massey et al., 2008).

Experiences with a high degree of emotional salience are better remembered than emotionally neutral events and this enhancement of memory may be mediated through actions of the lateral amygdala (Roozendaal et al., 2008). The strong reciprocal connectivity between the lateral amygdala and the perirhinal cortex (Pitkänen et al., 2000; Kajiwara et al., 2003) provides an anatomical basis by which the lateral amygdala may profoundly influence mechanisms of recognition memory. Indeed, inputs from the lateral nucleus of the amygdala (Furtak et al., 2007) directly influence perirhinal cortex activity and regulate synaptic transmission between the perirhinal cortex and the entorhinal cortex (Paz et al., 2006). However, the receptor mechanisms by which the lateral amygdala might modulate synaptic plasticity, and learning and memory, have not yet been fully elucidated.

A recent publication has described the mechanisms of long-term potentiation (LTP) at amygdala−perirhinal synapses (Laing and Bashir, 2014). However, the mechanisms of long-term depression (LTD) induction at the input from the lateral amygdala to the perirhinal cortex have not been investigated. These mechanisms are important to determine since LTD-like mechanisms are critical in perirhinal cortex-dependent recognition memory (Warburton, et al., 2003; Griffiths, et al., 2008; Massey, et al., 2008). Therefore, the aim of this study was to examine in an *in vitro* brain slice preparation the receptors required for amygdala−perirhinal LTD and whether there is an overlap between the mechanisms required for amygdala−perirhinal LTP and intracortical plasticity.

## Materials and Methods

Juvenile male Lister Hooded rats 30-35 d of age (80-100 g; Harlan Laboratories) were maintained on a 12 h light/12 h dark cycle (dark phase during normal daylight). All animal procedures are performed according to the regulation of authors’ university’s animal care committee.

### Slice preparation and electrophysiology

Animals were anesthetized with an isoflurane/oxygen mixture and decapitated, and the brain was rapidly removed. The brain was placed in ice-cold artificial CSF (aCSF; bubbled with 95% O_2_/5% CO_2_), which comprised the following (in mM): 124 NaCl, 3 KCl, 26 NaHCO_3_, 1.25 NaH2PO_4_, 2 CaCl_2_, 1 MgSO_4_, and 10 D-glucose. The olfactory bulb, the cerebellum, and the brain stem were removed. A horizontal cut was made anterior to posterior on the ventral surface of the brain. This created a flat surface by which the brain was glued, ventral side down, to the vibroslice stage. Ventral slices (400 µm thick) contained area 35 of perirhinal cortex and the lateral nucleus of the amygdala; the most dorsal slice also contained area 36. Slices were stored submerged in aCSF (20–25 °C) for at least 1 h before transferring to the recording chamber. A single slice was placed in a submerged recording chamber and perfused with aCSF (30-32 °C; flow rate 2 ml/min).

### Whole-cell recordings

Recording electrodes (pulled on Sutter Instruments P-87 puller) were filled with intracellular solution [(in mM): CsMeSO_4_ 130, HEPES 10, EGTA 0.5, MgATP 4, NaGTP 0.3, QX-314-Cl^-^ 5, NaCl 8 (280-300 mOsm, pH 7.2)] and were of 2.5-4 MΩ resistance. Excitatory postsynaptic currents were evoked by a bipolar stimulation electrode (frequency of simulation: 0.033 Hz) placed in the lateral amygdala (designated the LA-PRh input). Whole-cell recordings were made from layer II/III neurons and, unless otherwise stated, the cells were voltage clamped at −70 mV during recording. To induce LTD, a low-frequency stimulation protocol (LFS; 200 stimuli, 1 Hz) was paired with depolarisation of the cell to −40 mV by injection of Direct current (DC) through the recording pipette. Recordings were made using an Axopatch 200B amplifier (Molecular Devices). Amplitudes of the evoked EPSCs were measured and expressed relative to the normalised baseline (see Analysis section). The data was acquired using WinLTP (v2.01). Recordings were filtered at 5 kHz and digitized at 20 kHz (Digidata 1322A; Molecular Devices). Only cells that had a series resistance (*R*_S_) <25 MΩ and <25% change in the *R*_S_ during the course of the experiment were included for analysis. Postsynaptic blockade of NMDARs was achieved by adding (+)-MK-801 maleate (3 mM) to the intracellular filling solution. To ensure MK-801 blockade of postsynaptic NMDARs, the cells were briefly depolarised to −10 mV between stimulations to relieve the magnesium block and aid in MK-801 entry. All cells were patched for a minimum of 20 min prior to the induction of LTD. For experiments recording NMDAR EPSCs, the slice was continuously perfused with NBQX (5 μM) and picrotoxin (50 μM).

### Pharmacological agents

Unless otherwise stated, all compounds were obtained from Tocris Bioscience and were prepared as stock solutions (1-10 mM) by dilution in double-distilled water and aliquoted for storage at −20 °C. The following compounds were diluted in aCSF and bath applied at the following concentrations: RO 25-6981 maleate (1 µM), picrotoxin (50 μM), NBQX (5 μM), D-AP5 (50 µM), propranolol hydrochloride (500 nM), verapamil hydrochloride (20 µM), TCN-201 (10 µM), NVP-AAM077 (200 nM), and L-689,560 (5 μM).

### Analysis

The peak amplitude of EPSCs were normalised to an initial 10 min baseline. When experiments were pooled together to generate summary graphs, the mean and the SEM (±) were calculated from the pooled data sets. For statistical analysis, the last 10 min of the baseline were compared to the last 10 min of the protocol. Therefore, statistics based on LTD refer to statistical significance 30 min after LFS. For comparison between two different conditions, an unpaired student’s *t* test was performed. For this, the normalised pooled data was used to run statistical comparisons between the two different groups. To study a before and after effect (e.g., baseline vs LFS) within the same group, a paired Student’s *t* test was performed. In all cases a superscript letter follows the associated p value. This relates to the statistical figure on page 7. In all experiments, *N* is equal to the number of rats euthanized for that experiment. The paired-pulse facilitation was determined by expressing the amplitude of the second response as a proportion of the amplitude of the first response. Statistics and graphs were generated using SigmaPlot V12.5. The software was also used to test for normality, using the Shapiro−Wilk test.

## Results

### LTD at the LA−PRh input relies on activation of NMDARs

**Figure 1:**
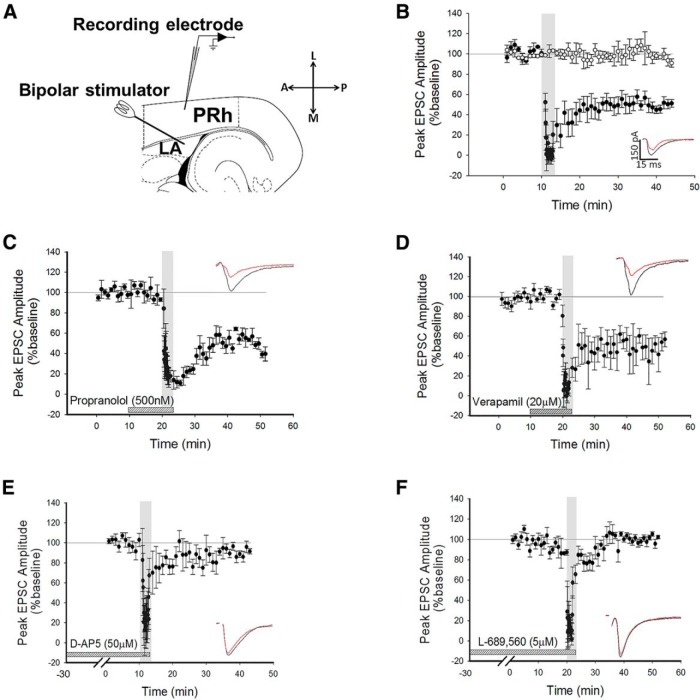
LTD is NMDAR-dependent, but is not dependent on β-ADRs or VGCCs. ***A***, Schematic diagram highlighting the recording and stimulating positions within the horizontal slice preparation with LA and PRh highlighted. L, Lateral; A, anterior; M, medial; P, posterior. ***B***, Pooled data showing induction of LTD by LFS (200 pulses at 1 Hz, black circles) during application of a holding potential to −40 mV. Intercalated control experiments are presented as white circles. Inserts here and all graphs show response traces during baseline (black) and following manipulation (red). In all cases, the scale bar dimensions remain the same (150 pA by 15 ms). Dotted horizontal lines act as a visual aid to the 100% baseline and the gray vertical bars represent the period of LFS delivery. ***C***, Pooled data highlighting that blockade of β-ADRs with propranolol did not prevent the induction of LTD. ***D***, Pooled data showing that LTD induction was not dependent on VGCCs. ***E***, Pooled data highlighting that bath application of D-AP5 prevented the induction of LTD. ***F***, Pooled data to show that application of L-689,560 prevented the induction of LTD.

Whole-cell recordings were made from Layer II/III neurons in perirhinal cortex (PRh) and voltage-clamped at −70 mV, unless otherwise stated. Stimuli were delivered every 30 s to the lateral amygdala (LA) to evoke EPSCs in the recorded cell (Fig. 1*A*). After obtaining a stable baseline of 10 min, 200 stimuli were delivered at 1 Hz (low-frequency stimulation; LFS) while maintaining a holding potential of −40 mV. The use of this protocol consistently produced robust LTD (depression to 50 ± 5% of baseline, *p* = 0.0045^a^, *N* = 6; Fig. 1*B*). To ensure that the conditions of recording did not produce rundown of synaptic transmission, control experiments were intercalated with the LTD experiments (Fig. 1*B*, white circles). Under these conditions, no significant reduction in baseline was observed (100 ± 6%, df = 5, *p* = 0.6, *N* = 6, Fig. 1*B* ^b^).

It has been demonstrated that beta adrenoceptors (β-ADRs) and voltage-gated calcium channels (VGCCs) are required for the induction of LTP at the LA−PRh input (Perugini et al., 2012). In contrast to the mechanisms of amygdala−perirhinal LTP, we observed that the induction and maintenance of LTD was not blocked by the β-ADR antagonist propranolol (depression to 39 ± 9% of baseline, df = 3, *p* = 0.01^c^, *N* = 4; Fig. 1*C*) nor by the VGCC inhibitor verapamil (52 ± 12%, df = 6, *p* = 0.0089^d^, *N* = 7; Fig. 1*D*). Since the perirhinal cortex can utilise NMDARs for the induction of LTD (Cho et al., 2000), we questioned whether amygdala−perirhinal LTD may similarly be NMDAR-dependent.

Bath application of the nonspecific NMDAR antagonist (Monaghan and Jane, 2009) D-AP5 (50 µM) blocked the induction of LTD (90 ± 4%, df = 5, *p* = 0.16^e^, *N* = 6; Fig. 1*E*). To confirm this observation, a structurally dissimilar NR1-selective antagonist, L-689,560 (5 μM) (Yao and Mayer, 2006), was bath applied. This antagonist, which acts at the glycine-binding site of the NMDAR, also blocked the induction of LTD (99 ± 3%, df = 3, *p* = 0.41^f^, *N* = 4; Fig. 1*F*). These results demonstrated that the receptors required for the induction of LTD at the LA−PRh synapse are different to the mechanisms of induction of LTP at the LA−PRh input (Laing and Bashir, 2014).

### LTD at the LA−PRh input relies on presynaptic NMDARs

**Figure 2:**
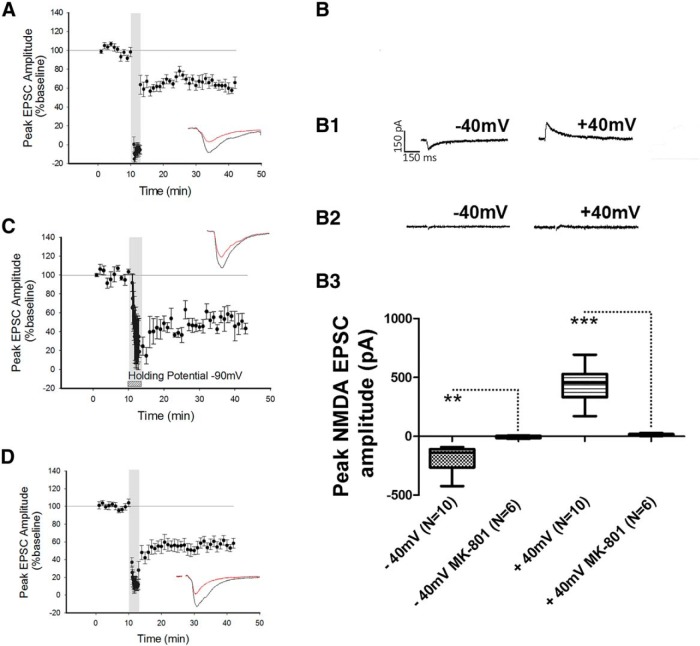
Blockade of postsynaptic NMDARs did not prevent the induction of LTD. ***A***, Application of the NMDAR pore-channel blocker MK-801 to the internal patch solution did not prevent LFS from inducing LTD. ***B***, Control experiment demonstrating that NMDAR currents could not be evoked when MK-801 was present in the internal patch solution. ***B1***, ***B2***, Traces of an NMDAR EPSC at −40 mV and +40 mV in the absence (***B1***) and presence (***B2***) of internal MK-801. ***B3***, Box plot demonstrating the peak amplitude of the NMDAR response in the presence and absence of MK-801 in the internal patch solution. ***C***, Holding the cell at −90 mV during the delivery of LFS did not prevent the induction of LTD. ***D***, Application of the calcium chelator BAPTA to the internal patch solution did not prevent the induction of LTD. **p ≤ 0.01, ***p ≤ 0.001.

Previous studies have shown that postsynaptic NMDARs are required for the induction of LTD at intraperirhinal synapses (Massey et al., 2004). We therefore examined whether the NMDARs required for the induction of LTD at the LA−PRh input were postsynaptically or presynaptically located. To specifically block postsynaptic NMDARs, MK-801 (3 mM) was added to the internal patch solution and cells were allowed a minimum of 20 min to equilibrate prior to LTD induction. MK-801 displays an IC_50_ of 0.01 µM for both NR1/NR2A and NR1/NR2B; we therefore expected this compound to inhibit all postsynaptic NMDARs (Paoletti and Neyton, 2007). However, under these conditions, LTD was not blocked (depression to 63% ± 5%, df = 8, *p* = 0.029^g^, *N* = 9; Fig. 2*A*) and the magnitude of LTD was not significantly different (*p* = 0.92^h^) from control LTD (Fig. 1*B*). To ensure that under the conditions of our experiments, NMDA EPSCs were blocked by MK-801, cells were patched with internal MK-801 while AMPAR and GABAR-mediated transmission were blocked with bath application of NBQX and picrotoxin, respectively; in these experiments, no NMDA EPSCs could be observed (*N* = 6; Fig. 2*B*,*B2*,B^3^). In separate control experiments, NMDA EPSCs were consistently evoked by LA stimulation in the absence of internal MK-801 (Fig. 2*B*,*B1*,B^3^). Statistical analysis of −40 mV (*N* = 10) against −40 mV in the presence of intracellular MK-801 (*N* = 6) yielded statistical significance (*p* = 0.015^i^). Similarly, the comparison of NMDAR peak amplitude between +40 mV (*N* = 10) and +40 mV in the presence of MK-801 (*N* = 6) was significant (*p* ≤ 0.0001^j^).

To further examine a possible role for postsynaptic NMDARs in LTD at the LA−PRh input, neurons were hyperpolarised by DC injection to −90 mV during the delivery of LFS to sustain the Mg^2+^ block of the NMDAR pore. However, this manipulation did not prevent the induction of LTD (50% ± 8.4%, df = 2, *p* = 0.028^k^, *N* = 3; Fig. 2*C*). To further corroborate our findings, an experiment was performed in which the calcium chelator BAPTA (3 mM) was included in the internal patch solution. For these experiments, the LFS was paired with a holding command of −40 mV. Under these conditions, we still observed that LFS induced LTD (49% ± 7%, df = 9, *p* ≤ 0.0001^l^, *N* = 10; Fig. 2*D*).

Together, the above results demonstrated that LTD at the LA−PRh input did not require postsynaptic NMDARs, and additionally indicated that a postsynaptic increase in calcium (from some source other than NMDARs) was not required for LTD. This suggested that the mechanism of LTD at LA−PRh synapses most likely relies on presynaptic NMDARs and is therefore different to LTD at intracortical PRh synapses (Massey et al., 2004).

### NR2A-containing NMDARs are necessary for LTD

**Figure 3:**
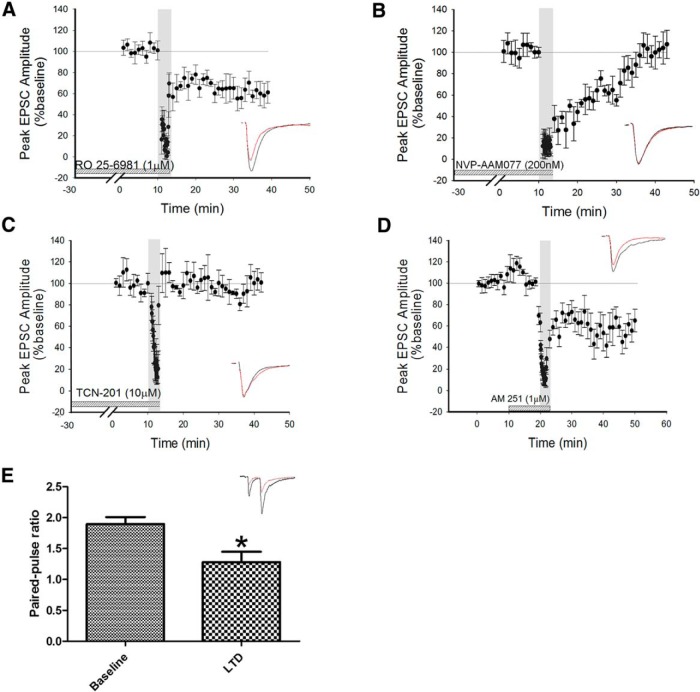
LTD induction relies on NR2A- but not NR2B-containing NMDARs. ***A***, Bath application of the NR2B-selective antagonist RO 25-6981 did not prevent the induction of LTD. ***B***, Bath application of the NR2A-selective antagonist NVP-AAM077 prevented the induction of LTD. ***C***, Bath application of the NR2A-selective antagonist TCN-201 prevented the induction of LTD. ***D***, Bath application of the CB1R antagonist AM 251 did not prevent the induction of LTD. ***E***, Paired-pulse facilitation is observed at the LA−PRh input during baseline recording and 30 min post-LFS. However, the facilitation is reduced by the delivery of the LFS protocol. *p < 0.05.

Previous studies have suggested a role for presynaptic NR2B-containing NMDARs in the induction of LTD in rat neocortex (Sjöström et al., 2003). Therefore, we investigated whether the induction of LA−PRh LTD was sensitive to blockade of NR2B-containing NMDARs. For this we chose RO 25-6981, which shows a >1000-fold selectivity for NR2B- over NR2A-containing NMDARs, and has not been demonstrated to inhibit the NR2A subunit until a concentration of 10 µM is reached (Volianskis et al., 2013). We found that the bath application of the NR2B-selective antagonist RO 25-6981 (1 μM) did not prevent the induction of LTD (64% ± 10%, df = 4, *p* = 0.012^m^, *N* = 5; Fig. 3*A*). The next obvious step was to determine whether NR2A-containing NMDARs might be required for LTD at the LA−PRh synapse. Blockade of NR2A-containing NMDARs was achieved by the bath application of NVP-AAM077 at a concentration (200 nM) shown to be selective for NR2A- over NR2B-subunit-containing NMDARs (Bartlett et al., 2007). Indeed, we observed that the pharmacological blockade of NR2A-containing NMDARs prevented the induction of LTD (98 ± 12%, df = 4, *p* = 0.054^n^, *N* = 5; Fig. 3*B*). To confirm a role for NR2A-containing NMDARs in LTD, we repeated the experiment with a structurally dissimilar NR2A antagonist. For this, we utilised TCN-201 (10 μM). This compound displays a >1000-fold selectivity for NR1/NR2A over NR1/NR2B, and at a concentration of 10 µM has no reported antagonistic effect on NR2B-containing NMDARs (Edman et al., 2012; Hansen et al., 2014). We found that the application of TCN-201 also prevented the induction of LTD (94 ± 8%, df = 3, *p* = 0.22^o^, *N* = 4; Fig. 3*C*).

Overall, our data suggested that the induction of LTD at the LA−PRh input relies on the activation of presynaptic NR2A-containing NMDARs. Previous work demonstrating a role for presynaptic NMDARs in the visual cortex has suggested that activity of cannabinoid CB1 receptors, coincident with presynaptic NMDAR activity, is essential for LTD (Sjöström et al., 2003). However, under the conditions of our experiments, postsynaptic BAPTA did not prevent LTD (Fig. 2*D*), and therefore, it is unlikely that a retrograde signal from the postsynaptic cell (that relies on postsynaptic calcium) is involved in the induction of LTD at the LA−PRh synapse. Nevertheless, we examined whether there may be a role for cannabinoid signaling by using the CB1 antagonist AM251 (1 μM). However, we found that the induction of LTD was not blocked by bath application of AM251 (71 ± 12%, df = 4, *p* = 0.034^p^, *N* = 5; Fig. 3*D*). The structurally dissimilar CB1 receptor antagonist LY320135 (5 μM) also had no effect on the induction of LTD (58 ± 8%, df = 2, *p* = 0.044^q^, *N* = 3; data not shown). Therefore, retrograde signalling (at least via cannabinoid receptors or triggered by a postsynaptic rise in calcium) is not required to be coincident with presynaptic NMDAR activation in order for LTD to be induced at the LA−PRh synapse.

Finally, to gain some insight into the locus of expression of LTD, we examined how LTD affects paired-pulse facilitation (PPF). We found that 30 min following the induction of LTD, PPF (1.3 ± 0.15) was significantly reduced compared to basal conditions (1.9 ± 0.1, *N* = 7, *p* = 0.023^r^, df = 6, *N* = 7; Fig. 3*E*;Table 1).

**Table 1:** Statistical table

	Data structure	Type of test	*p* value
a	Normal distribution	Paired *t* test	0.0045
b	Normal distribution	Paired *t* test	0.6
c	Normal distribution	Paired T test	0.01
d	Normal distribution	Paired *t* test	0.0089
e	Normal distribution	Paired *t* test	0.16
f	Normal distribution	Paired *t* test	0.41
g	Normal distribution	Paired *t* test	0.029
h	Normal distribution	Unpaired *t* test	0.92
i	Normal distribution	Unpaired *t* test	0.0015
j	Normal distribution	Unpaired *t* test	≤0.0001
k	Normal distribution	Paired *t* test	0.028
l	Normal distribution	Paired *t* test	≤0.0001
m	Normal distribution	Paired *t* test	0.012
n	Normal distribution	Paired *t* test	0.054
o	Normal distribution	Paired *t* test	0.22
p	Normal distribution	Paired *t* test	0.034
q	Normal distribution	Paired *t* test	0.044
r	Normal distribution	Unpaired *t* test	0.023

## Discussion

The amygdala has an established role in fear conditioning (Blair et al., 2001; Schafe et al., 2001; Maren and Quirk, 2004; Johansen et al., 2010), with information for these events being stored in the amygdala as a result of synaptic plasticity (Rogan et al., 1997; Fanselow and LeDoux, 1999; Blair et al., 2001). However, a less well defined, yet highly important role, of the amygdala is its ability to influence other forms of memory, particularly when the learning episode has a degree of emotional salience (Paz et al., 2006). The mechanisms by which the lateral amygdala modulates LTP in the perirhinal cortex have only recently been described (Laing and Bashir, 2014), and this paper expands our understanding of these mechanistic processes by studying the receptor mechanisms required for amygdala−perirhinal LTD.

We observed that LTD between the lateral amygdala and layer II/III of the perirhinal cortex is NMDAR-dependent and does not rely on β-ADRs or VGCCs. This is in contrast to LTP between the lateral amygdala and perirhinal cortex, which is NMDAR-independent and β-ADRs/VGCC-dependent (Perugini et al., 2012; Laing and Bashir, 2014). Therefore, the mechanisms of induction for LTP and LTD at the same input from the lateral amygdala to the perirhinal cortex are very different.

Surprisingly, we found that although the induction of LTD relied on the activation of NMDARs, postsynaptic mechanisms do not appear to play a role in LTD induction at the LA−PRh input. Thus, neither a high concentration of BAPTA in the postsynaptic cell, nor blockade of postsynaptic NMDARs by MK-801 loaded in the patch pipette, nor hyperpolarising the postsynaptic neuron to maintain the Mg^2+^ block of the NMDAR channel resulted in a block of LTD. Therefore, a simple conclusion from these results is that the NMDARs crucial for the induction of LTD are presynaptically located.

A recent study in hippocampus (Nabavi et al., 2013) reported that the competitive NMDAR antagonist AP5 prevented the induction of LTD but that MK-801 or glycine site antagonists did not block LTD and suggested a metabotropic function of NMDARs in LTD (but see Babiec et al., 2014). While these results are similar in some respects to our findings, there is an important difference: in our study, the glycine site antagonist blocked the induction of LTD. Therefore, in agreement with Babiec et al. (2014), NMDAR activation in our study most likely relies on canonical NMDARs, rather than metabotropic NMDARs.

Several studies have provided evidence for the importance of presynaptic NMDARs in the induction of LTD (Sjöström et al., 2003; Lien et al., 2006; Corlew et al., 2007; Brasier and Feldman, 2008; Rodríguez-Moreno and Paulsen, 2008). It has been suggested that the presynaptic NMDARs responsible for LTD in the visual cortex are NR2B-subunit containing. However, in our current work, the NR2B antagonist RO 25-6981 did not block LTD at the LA−PRh input. Interestingly, a role for retrograde cannabinoid signaling following a postsynaptic rise in calcium was postulated to be important for induction of LTD, where NR2B-NMDARs were shown to be required (Sjöström et al., 2003). Though, in our study, inhibitors of CB1 receptors did not affect LTD, and neither did LTD rely on a rise in postsynaptic calcium. It is unlikely, therefore, that cannabinoids generated postsynaptically, or from other sources, are necessary for the induction of LTD.

In contrast to the above studies in visual cortex, we found that two different antagonists of NR2A-containing NMDARs blocked the induction of LTD. Therefore, it is most likely that presynaptic NR2A-containing NMDARs are responsible for the induction of LTD at the LA−PRh input. These results are in contrast to the postsynaptic NR2B-NMDAR mechanisms for LTD that have been described at intracortical PRh synapses (Massey et al., 2004) and that are considered to be essential for visual recognition memory (Griffiths et al., 2008). Overall, this demonstrates that LTD at different inputs to the perirhinal cortex can rely on very different receptor mechanisms for the induction of plasticity.

Indeed, it has been demonstrated that LTD at two different inputs onto layer II neurons in the visual cortex relied on two different mechanisms (Larsen et al., 2014). Activation of presynaptic NR2A-containing NMDARs were shown as necessary for LTD at the parallel fiber−purkinje cell synapse, and it has been suggested that the role of these presynaptic receptors is to detect the temporal patterns of presynaptic action potential firing. Thus, it is possible that intracortical PRh synapses, that encode recognition memory, rely on Hebbian integration mechanisms but that LA−PRh synapses rely on the temporal structure of presynaptic action potential firing to modulate recognition memory.

A reasonable question to ask is whether NR3-containing NMDARs may have any role in the LTD observed in this study, considering that these receptors have demonstrated importance in presynaptic plasticity (Corlew et al., 2008). To date, we know very little about this synapse, and we know, overall, little about presynaptic mechanisms and physiological functions of NR3-containing NMDARs. However, in this study, we believe NR3-containing NMDARs are unlikely to contribute to the LTD observed (either as NR1/NR3 or as NR1/NR2/NR3).

A comparison of NR1 or NR2 versus NR3 shows ∼24% sequence homology, with structural differences that suggest a very different pharmacological binding profile (Monaghan and Jane, 2009). As an example, L689,560, which blocked LTD, is strongly selective for NR1- over NR3-containing NMDARs binding with nanomolar affinity (Yao and Mayer, 2006). Also, TCN-201 (which blocked LTD) inhibition is mediated by residues from both NR1 and NR2A subunits (Edman et al., 2012) and can only inhibit NMDARs via interaction with the dimer interface between NR1/NR2A. It is also worth highlighting that NR3 expression starts to decrease around the time of eye opening (Bendová et al., 2009), with low immunohistochemical expression of NR3 past P21, and remaining low into adulthood (Sucher et al., 1995). Further, *in vitro* electrophysiology has observed no significant NR3 contribution to NMDA-activated currents past P11−P13 (Sasaki et al., 2002).

The work presented here demonstrates the complexity of plastic mechanisms between the lateral amygdala and the perirhinal cortex. While underpinning some of the key receptor mechanisms involved in the induction of LTD at this synapse, we leave further avenues of research available for future studies to establish. Though it is beyond the scope of this paper to also study the mechanisms by which LTD expression occurs, our observation that paired-pulse facilitation is reduced at this synapse following LTD at least highlights a presynaptic role for the expression of LTD. However, the change in the PPF following the induction of LTD was unexpected, as a presynaptic mechanism for LTD would be expected to decrease transmitter release, which is classically associated with an increase in PPF.

One possibility that explains our findings is that the release of glutamate following the first stimulus binds presynaptic NMDARs, so that during the second stimulus NMDARs are activated and lead to an increase in the presynaptic calcium entry that contributes significantly to the PPF. A presynaptic decrease in transmitter release, following LTD, would then result in less activation of NMDARs during the second stimulus, reduced calcium entry, and therefore, a decrease in PPF. In support of this possibility, Berretta and Jones (1996) observed in the entorhinal cortex an approximate 20% reduction in paired-pulse facilitation during superfusion with D-AP5 (while blocking postsynaptic NMDARs with MK-801 in the patch pipette).

Overall, in this paper we provide fundamental insights into the basic mechanisms of LTD induction at this synapse. We have demonstrated that LTD between the lateral amygdala and layer II/III of the perirhinal cortex is induced presynaptically via NR2A-containing NMDARs, and we expand on current literature, which demonstrates the receptor mechanisms of LTP between these two regions (Perugini et al., 2012; Laing and Bashir, 2014). The combination of this body of research demonstrates that the receptors required for LTD and LTP at the LA−PRh input are NMDAR-dependent and NMDAR-independent, respectively. Potentially, the requirement for β-ADRs in LTP (Laing and Bashir, 2014) but not in LTD, as demonstrated here, suggests that emotional stimuli will impact synaptic events in a differential manner.

## Synthesis

The decision was a result of the Reviewing Editor Robert Kalb and the peer reviewers coming together and discussing their recommendations until a consensus was reached. A fact-based synthesis statement explaining their decision and outlining what is needed to prepare a revision is listed below. The following reviewers agreed to reveal their identity: Serena Dudek and Akiva Cohen

Your manuscript has been sent out to two reviewers and as you can see by their comments, they are potentially interested in your work there there are concerns. The major issue raised by Reviewer #1 concerns the locus of LTD. This reviewer would like to see the results of paired-pulse facilitation/ratio. If these synapses don't facilitate, as is sometimes the case with different cortical synapses, then an analysis of the CoV in their existing data sets could give some hints. Reviewer #2 has significant concerns about the definition of LTD and the statistical methods applied. You will need to direct address all the criticisms raised but play particular attention to these two sets of comments. Once you have done this and re-submitted the ms., it will be sent out for re-review.

### Review # 1:

This manuscript details some of the mechanisms underlying long-term synaptic depression (LTD) of amygdala-> perirhinal cortex synapses. The authors present strong evidence that the LTD, while NMDA receptor dependent, is distinct mechanistically from other forms of cortical LTD in that it appears to rely on presynaptic NR2A containing NMDARs. They further determined that the depression does not require beta adrenergic receptors, voltage sensitive calcium channels, NR2B containing receptors, or CB1 cannabinoid receptors. Although far from being definitive experiments (such as knock out of NR2As in the amygdala, for example), the study provides solid pharmacological evidence in support of the authors' conclusions. The experiments also appear to be well executed, analyzed, and presented. Overall the manuscript is well written and does a nice job of explaining the importance of studying this particular synapse. I have a few suggestions for improving the manuscript, listed below.

1) The authors did not address whether the expression of the LTD was presynaptic (as is its induction). Is paired-pulse facilitation observed at this synapse? If so, it could be useful to test before/after LTD as one measure of presynaptic plasticity. Also, if the data on the Coefficient of Variation is available for analysis, it could be similarly informative on this topic.
2) The authors should discuss the possibility, if any, that NR3A containing receptors are underlying this plasticity, especially as these receptors have been shown to be important for presynaptic plasticity (Corlew, et al.) and it would seem those receptors would be very sensitive to the L-689,650. Also, if there is data known on the selectivity of the inhibitors of NR2A over NR3A, please provide. Also, citations on the selectivity of some of these drugs would be appropriate (TCN-201, L-689,650, for example).
3) In the significance statement, the authors write, "... the first demonstration of the receptors required for amygdala-perirhinal LTD", and " a role for NMDARs in amygdala-...." are redundant statements and can be combined in some way. Also, in the same sentence, they state, "the mechanisms described here are different to amygdala-perirhinal LTP...", which is rather obvious (LTP vs. LTD). I think the authors could clarify this by adding, "which are both induced postsynaptically.", or something to that effect.
4) First sentence of Introduction: Most of the citations on visual recognition memory are from work performed in primate, with only one relevant to the study in rat. Please distinguish the two. Also, a citation is needed for the reliance on lasting decrements in neuronal activation (same sentence).

### Review #2:

This report examines the mechanisms underlying long-term depression (LTD), at the amygdala-perirhinal synapse. Understanding this mechanism is important because it could provide insight into how visual recognition memory expressed in the perirhinal cortex can be modified with emotional salience by amygdala activation.

LTD was induced by stimulation of the lateral amygdala using low-frequency tetanic stimulation protocol (LFS) and performing whole-cell recordings from single cells in layer II/III of the perirhinal cortex. The amplitude of the excitatory post-synaptic currents (EPSCs) were recorded every 30 s for an initial ten minute baseline and subsequently after LFS for an additional 30 minutes. The presence of LTD was determined by calculating the percent difference of the average of the last ten normalized post-LFS EPSC amplitudes compared to the average of the last ten normalized baseline EPSC amplitudes. After establishing LTD in the control condition, LTD was assessed in the presence of various pharmacological blockers to determine the mechanism(s) contributing to LTD. Bath application of the NMDAR antagonist D-AP5 blocked LTD, signifying that NMDARs contribute to this plasticity at the lateral amygdala-perirhinal synapse. Moreover, blockage of post-synaptic NMDARs (NMDAR antagonist MK-801 added to recording pipette solution) and hyperpolarization of the post-synaptic cell did not prevent LTD, indicating that LTD is mediated by pre-synaptic NMDARs. Further experiments demonstrated that the presynaptically mediated LTD was not dependent on either a post-synaptic calcium rise (by addition of BAPTA to recording solution) or a retrograde endocannabinoid signal (bath application of CB1 antagonist AM251). To specify the composition of the pre-synaptic NMDARs, bath application of NVP-AAM077, a selective antagonist for NR2A-containing NMDARs, hindered LTD. Overall, these experiments suggest that LTD at the lateral amygdala-perirhinal cortex synapse relies on NR2A-containing, pre-synaptic NMDARs.

Major Considerations

1. The statistics performed in this paper are meaningless for several reasons: First, the authors fail to define a criterion for what they consider LTD. They consider any significant difference between the baseline and the post-LFS EPSC amplitudes to be LTD, however, just because there is a significant difference does not mean there is necessarily LTD. This is of crucial importance seeing as a significant difference could be due to other factors such as current "run down" from protracted whole-cell recordings (approximately for 60 min). Therefore they need to define a criterion for what is considered LTD to give meaning to their significant differences. Second, it is not clear what is being statistically compared. The number of samples "N" was defined as the number of animals, but this only works if only 1 brain slice was used for each animals. This needs to be stated. If the authors use more than one slice/animal then they need to state what is being compared i.e., number of slices or animals. Third, the number of animals (N) appears to be relatively small in many experiments.
2. The table included in the manuscript is not correct. It does not contain power calculations. If the p-values listed were determined by a power calculation, the power value and minimum sample size to achieve statistical power need to be listed. How was normality of the data actually assessed?

## Author Response

**Responses to Reviewer No. 1**

**The authors did not address whether the expression of the LTD was presynaptic (as is its induction). Is paired-pulse facilitation observed at this synapse? If so, it could be useful to test before/after LTD as one measure of presynaptic plasticity. Also, if the data on the Coefficient of Variation is available for analysis, it could be similarly informative on this topic.**

During this study we focussed on providing insight into the mechanisms of LTD induction. However, during these experiments we did perform a preliminary set of paired-pulse experiments, though, as you will see below, these data were somewhat surprising. However, in light of the referee’s comments the data has been included (Figure 3E), with appropriate changes to the text.

***Results***: *“Finally, to gain some insight into the locus of expression of LTD we examined how LTD affects paired pulse facilitation (PPF). We found that 30-minutes following the induction of LTD PPF (1.3 ± 0.15) was significantly reduced compared to basal conditions (1.9 ± 0.1, N=7, P=0.023, df=6, N=7, Figure 3E).”*
***Discussion****: “Though it is beyond the scope of this paper to also study the mechanisms by which LTD expression occurs, our observation that paired-pulse facilitation is reduced at this synapse, following LTD, at least highlights a presynaptic role for the expression of LTD. However, the direction of change in PPF following the induction of LTD was unexpected, as a presynaptic mechanism for LTD would be expected to decrease transmitter release, which is classically associated with an increase in PPF.*
  One possibility, to explain our findings, is to suggest that the release of glutamate, following the first stimulus, binds presynaptic NMDARs, so that during the second stimulus NMDARs are activated and leads to an increase in the presynaptic calcium entry that contributes significantly to the PPF. A presynaptic decrease in transmitter release, following LTD, would then result in less activation of NMDARs during the second stimulus, reduced calcium entry, and therefore, a decrease in PPF. In support of this possibility, Berretta and Jones (1996), observed in the entorhinal cortex an approximate 20% reduction in paired-pulse facilitation during superfusion with D-AP5 (whilst blocking postsynaptic NMDARs with MK-801 in the patch pipette).”
**Figure legend 3: “E:** Paired-pulse facilitation is observed at the LA-PRh input during baseline recording and 30-minutes post-LFS. However, the facilitation is reduced by the delivery of the LFS protocol. “

**The authors should discuss the possibility, if any, that NR3A containing receptors are underlying this plasticity, especially as these receptors have been shown to be important for presynaptic plasticity (Corlew, et al.) and it would seem those receptors would be very sensitive to the L-689,650. Also, if there is data known on the selectivity of the inhibitors of NR2A over NR3A, please provide.**

The reviewer raises an interesting point regarding the role of NR3A-containing NMDARs, which we now address:

***Discussion****: “A reasonable question to ask is whether NR3-containing NMDARs may have any role in the LTD observed in this study, when considering that these receptors have demonstrated importance in presynaptic plasticity (Corlew et al., 2008). To date we know very little about this synapse, and we know, overall, little about presynaptic mechanisms and physiological functions of NR3-containing NMDARs. However, in this study we believe NR3-containing NMDARs are unlikely to contribute to the LTD observed (either as NR1/NR3 or as NR1/NR2/NR3).”*
  “A comparison of NR1 or NR2 vs NR3 shows ∼24% sequence homology, with structural differences that suggest a very different pharmacological binding profile (Monaghan and Jane, 2009). As an example L689,560, which blocked LTD, is strongly selective for NR1- over NR3-containing NMDARs, binding with nanomolar affinity (Yao and Mayer, 2006). Also, TCN-201 (which blocked LTD) inhibition is mediated by residues from both NR1 and NR2A subunits (Edman et al., 2012) and can only inhibit NMDARs via interaction with the dimer interface between NR1/NR2A. It is also worth highlighting that NR3 expression starts to decrease around the time of eye opening (Bendová et al., 2009), with low immunohistochemical expression of NR3 past P21, and remaining low into adulthood (Sucher et al., 1995). Further, in vitro electrophysiology has observed no significant NR3 contribution to NMDA-activated currents past P11-P13 (Sasaki et al., 2002).”

**Also, citations on the selectivity of some of these drugs would be appropriate (TCN-201, L-689,650, for example).**

As requested, citations on compound selectivity have been added to the manuscript.

**Results**: “Bath application of the non-specific NMDAR antagonist (Monaghan and Jane, 2009).”
**Results**: “To confirm this observation a structurally dissimilar NR1 selective antagonist, L-689,560 (5μM) (Yao and Mayer, 2006), was bath applied. This antagonist, which acts at the glycine-binding site of the NMDAR, also blocked the induction of LTD”
**Results**: “MK-801 displays an IC_50_ of 0.01µM for both NR1/NR2A and NR1/NR2B, we therefore expected this compound to inhibit all postsynaptic NMDARs (Paoletti and Neyton, 2007).”
**Results**: “For this we chose RO 25-6981, which shows a >1000-fold selectivity for NR2B- over NR2A-containing NMDARs, and has not been demonstrated to inhibit the NR2A subunit until a concentration of 10µM is reached (Volianskis et al., 2013).”
**Results**: “For this we utilised TCN-201 (10μM). This compound displays a >1000-fold selectivity for NR1/NR2A over NR1/NR2B, and at a concentration of 10µM has no reported antagonistic effect on NR2B-containing NMDARs (Edman et al., 2012, Hansen et al., 2014). “

**In the significance statement, the authors write, "... the first demonstration of the receptors required for amygdala-perirhinal LTD", and " a role for NMDARs in amygdala-...." are redundant statements and can be combined in some way.**

This has now been changed to *“We provide the first demonstration of the mechanisms underlying LTD at this synapse, which is dependent on presynaptically located NR2A-containing NMDARs.”*

**Also, in the same sentence, they state, "the mechanisms described here are different to amygdala-perirhinal LTP...", which is rather obvious (LTP vs. LTD). I think the authors could clarify this by adding, "which are both induced postsynaptically.", or something to that effect.**

Changed to “We also show a role for NMDARs in amygdala-perirhinal plasticity, and that the mechanisms described here are different to those of amygdala-perirhinal LTP (NMDAR independent) and intra-perirhinal plasticity (NMDAR dependent), both of which are induced postsynaptically.”

**First sentence of Introduction: Most of the citations on visual recognition memory are from work performed in primate, with only one relevant to the study in rat. Please distinguish the two. Also, a citation is needed for the reliance on lasting decrements in neuronal activation (same sentence).**

The macaque and rodent references have been separated, and a more extensive list of rodent work, which I found of importance during my studies, has been included.

*“The familiarity discrimination component of visual recognition memory requires the perirhinal cortex, as demonstrated in both macaque (Gaffan and Murray, 1992, Fahy et al., 1993, Suzuki et al., 1993, Suzuki, 1996, Ennaceur and Aggleton, 1997, Murray and Bussey, 1999) and rodent (Aggleton et al., 1997, Ennaceur and Aggleton, 1997, Ennaceur et al., 1997, Dix and Aggleton, 1999, Winters et al., 2004) models”*.

**Responses to Reviewer No. 2**

**A significant difference could be due to other factors such as current "run down" from protracted whole-cell recordings (approximately for 60 min). Therefore they need to define a criterion for what is considered LTD to give meaning to their significant differences.**

The reviewer raises an important consideration that prolonged single-cell recordings can lead to rundown in the response, which is not associated with the physiological element under observation. However, we do not believe that the reduction in baseline observed is due to rundown from the length of recording.

–The reduction in response amplitude (e.g. Figure 1B) occurs immediately following the LFS protocol, and the LTD induced is stable.
–Experiments are only performed after at least 10-minutes of stable baseline.
–In cases where LTD is blocked the response returns to baseline and the response remains stable for a further 30 minutes (e.g. Figure 1E, 1F, 3B, 3C).

Finally, we include control experiments that were interleaved with the experiments in Figure 1B. The white circles relate to the interleaved controls showing that there is no ‘run-down’ under normal conditions. We apologise for not including these data in the original manuscript.

The following has now been added to the results section:

“To ensure that the conditions of recording did not produce rundown of synaptic transmission control experiments were intercalated with the LTD experiments (Figure 1B, white circles). Under these conditions no significant reduction in baseline was observed (100 ± 6%, df=5, P=0.6, N=6, Figure 1B^b^).”

**Second, it is not clear what is being statistically compared.**

Sentence changed for clarification,

**Methods:** “For statistical analysis the last 10-minutes of the baseline were compared to the last 10-minutes of the protocol. “
  Added to the text,
**Methods:** “Therefore, statistics based on LTD refer to statistical significance 30-minutes after LFS.”

**The number of samples "N" was defined as the number of animals, but this only works if only 1 brain slice was used for each animals. This needs to be stated. If the authors use more than one slice/animal then they need to state what is being compared i.e., number of slices or animals.**

Indeed, the reviewer is correct in their interpretation of the text. N= the number of animals used for that experiment. To explain, daily one animal was sacrificed and the resulting tissue prepared. Each experiment being undertaken would only be performed once with the resultant tissue from that animal. To help clarify any misunderstanding the text has been altered.

**Methods:** “In all experiments the N value is equal to the number of rats sacrificed for that experiment. “

**Third, the number of animals (N) appears to be relatively small in many experiments.**

We observe clear and consistent effects during our experiments and the error bars are within good reason to assure us of the validity of the results. As can be observed in the text the N values are well apportioned. In cases where the N value may appear “small” I would highlight that experiments where a significant result occurred were repeated numerous times, using structurally different antagonists, or recording conditions. Therefore we validate our data not just through the number of animals used but through the multiple approaches used to confirm the findings obtained.

**The table included in the manuscript is not correct. It does not contain power calculations. If the p-values listed were determined by a power calculation, the power value and minimum sample size to achieve statistical power need to be listed.**

This has been changed from “power” to “P-value”.

**How was normality of the data actually assessed?**

The following was added to the text.

**Methods:** *“Statistics and graphs of the data were generated using SigmaPlot V12.5. The software was also used to test for normality, using the Shapiro-Wilk test.”*
